# Supplementary material for: Potential Application of Protamine for Antimicrobial Biomaterials in Bone Tissue Engineering
Source: Int J Mol Sci. 2020 Jun 19;21(12):4368. doi: 10.3390/ijms21124368 (PMC7352774; doi:10.3390/ijms21124368)
Supplement: Supplementary file 1 [file ijms-21-04368-s001.pdf]

E. coli in the supernatant of an LB medium after 24 h culture was examined using a BacLight LIVE/DEAD staining kit by fluorescence microscopy. In the supernatant of HAp or protamine/HAp (125 μg·ml^{-1}), most cells were alive and showed the typical rod-like shape with a length of approximately 2 μm (Fig. S1a, b). Meanwhile, the length of E. coli in the supernatant of protamine/HAp (250-1000 μg·ml^{-1}) was about 2–10 times longer (Figs. S1c-e). These observations were in agreement with observations for the *E. coli* cultured on the protamine/HAp discs (Figs. 3 and 4). For protamine/HAp (2000 μg·ml^{-1}), no bacteria were observed in the supernatant (data not shown). These results demonstrated that a high dosage of protamine induced membrane disintegration and leading to cell death. No S. aureus was observed in the supernatant of all protamine/HAp. Therefore, we focused on the antibacterial properties of protamine/HAp on E. coli.

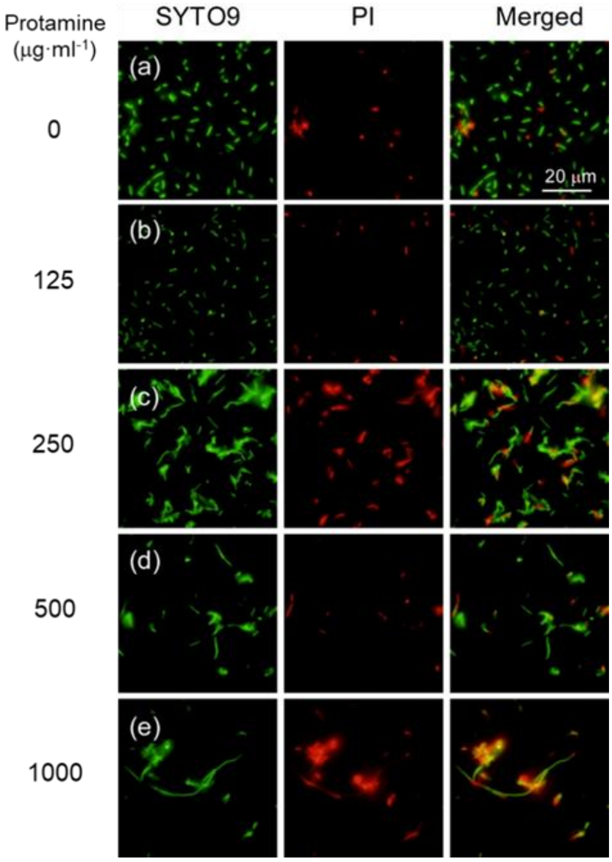

Figure S1. LIVE/DEAD staining of E. coli cells in the supernatants after 24 h culture. E. coli cells were cultured on various concentrations of protamine/HAp discs (a: 0 (HAp), b: 125, c: 250, d: 500, e: 1000 μg·ml^{-1}) for 18 h. Cells in the supernatants were stained using the LIVE/DEAD Bacterial Viability kit. High-dose protamine induced an aberrant morphology. Bar indicates 20 μm.

To investigate the morphology of the bacteria on the protamine/HAp discs in more detail, S. aureus on the discs were observed by SEM. No apparent changes or S. aureus were seen in their morphology, though adhered cells dramatically decreased (Fig. S2).

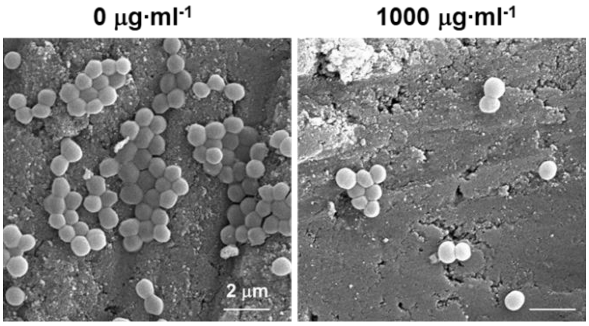

Figure S2. SEM images of S. aureus on the surface of HAp or protamine/HAp (1000) discs. S. aureus cultured on each disc for 24 h were observed by SEM. Bars indicate 2 μm.
